# Rapidly reconstructed CuCo_2_S_4_@Co–V–O–F nanocatalysts for efficient and stable overall water splitting in alkaline and seawater electrolysis†

**DOI:** 10.1039/d5ra03052h

**Published:** 2025-06-09

**Authors:** Boyao Zhang, Yinuo Zhao, Xin Li, Huiya Zhou, XinXin Zhao, Rongda Zhao, Fufa Wu

**Affiliations:** a School of Materials Science and Engineering, Liaoning University of Technology Jinzhou Liaoning 121000 P. R. China Rongdazhaoln@126.com

## Abstract

The strategic construction of bifunctional electrocatalytic electrodes integrating high activity and exceptional durability is critical for sustainable hydrogen generation through water and seawater splitting. Addressing challenges including sluggish reaction kinetics and chloride-induced corrosion in marine electrolyzers remains imperative. Mixed transition metal oxides/sulfides, particularly cobalt–vanadium-based composites, demonstrate superior electrocatalytic properties owing to their tunable electronic configurations, multivalent redox states, enhanced charge transfer capabilities, and abundant exposed active sites. Here, we have prepared CuCo_2_S_4_@Co–V–O–F. The electrode material is then calcined under argon protection, and a synergistic structural engineering and surface treatment adjustment strategy is adopted to construct nanostructures. The optimized catalyst exhibits remarkable bifunctional performance: low HER overpotentials of 87.8 mV (1 M KOH) and 95.5 mV (alkaline seawater) at −10 mA cm^−2^, coupled with OER overpotentials of 227.3 mV and 213.5 mV, respectively. Notably, the symmetric electrolyzer assembled with these nanowire arrays achieves an ultralow cell voltage of 1.796 V at 50 mA cm^−2^, demonstrating exceptional efficiency for overall water splitting while maintaining robust stability in corrosive saline media.

## Abstract 2

Here, we have successfully prepared the CuCo_2_S_4_@Co–V–O–F catalyst using a multi-step hydrothermal process. This catalyst showing low overpotentials. Under simulated seawater conditions, it achieves the overpotentials of 95.5 mV for HER.

## Graphical abstract

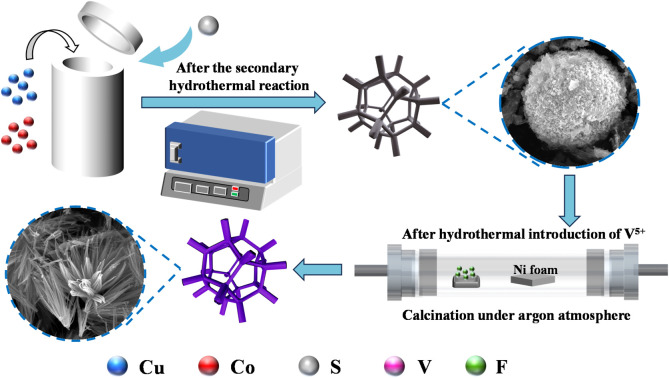

## 1. Introduction

In the past few years, the exploration of green and renewable energy as a sustainable alternative to environmentally harmful and finite fossil fuels has become a major global focus.^1–3^ In this context, among potential clean energy sources, hydrogen is considered a promising candidate for future energy applications due to its high energy density and zero-carbon emissions.^4,5^ Electrocatalytic water splitting represents a key technology in efficient energy conversion and storage systems. This process involves two half-reactions: the oxygen evolution reaction (OER) at the anode and the hydrogen evolution reaction (HER) at the cathode. The overall efficiency of water electrolysis is largely determined by the coupled kinetics of these two reactions. Since this thermodynamically non-spontaneous process requires external electrical energy input to overcome the Gibbs free energy barrier (Δ*G* > 0), minimizing the overpotential brings the operating voltage closer to the theoretical decomposition voltage of 1.23 V, thereby improving energy efficiency.^6–10^ It is known that there is an inevitable competition between oxygen evolution reaction (OER) and chlorine evolution reaction (CER) in seawater decomposition. These two reactions work together in the electrolysis process, resulting in an unsatisfactory overall decomposition voltage for a wide range of applications.^11–13^ Meanwhile, the O–H bond cleavage and O–O bond formation in the OER process involve complex multi electron transfer steps, resulting in slow kinetics of the reaction and further increasing the difficulty of achieving efficient seawater electrolysis.^14–16^ To accelerate its sluggish kinetics and adopt feasible electron transfer efficiency for hydrogen energy production, catalysts are necessary.^17,18^ Currently, the most effective electrocatalysts for OER are noble metal oxides such as RuO_2_ and IrO_2_, while Pt-based nanomaterials are the benchmark for HER. However, their scarcity and high cost pose significant barriers to large-scale industrial adoption of water splitting technologies. Therefore, developing highly active and stable non-noble metal catalysts has become a critical research focus in this field.^19–21^ Recent studies have shown that ternary transition metal sulfides, such as NiCo_2_S_4_, CuCo_2_S_4_, and MnCo_2_S_4_, due to their adjustable electronic structures, good conductivity, and interatomic synergistic catalytic effects, are considered promising electrode materials for water splitting. Among them, CuCo_2_S_4_ has been widely studied owing to the high catalytic activity of cobalt-based nanomaterials and the excellent conductivity of copper-based nanomaterials.^22–24^ However, the surface oxidation and corrosion of metal sulfides severely affect the water splitting performance and cycle stability of CuCo_2_S_4_.^25^ We chose to address these issues through composite formation and fluorination. It is known from relevant literature that vanadium resources are abundant and inexpensive, with excellent conductivity, and its atomic radius is close to Co.^26,27^ Moreover, the electronic and environmental structure provided by V's multiple valence states can effectively improve the deficiency of poor OER performance of materials. Changes in morphology and electronic structure brought about by bimetallic nanostructures also help expose more active sites, thereby enhancing the activity of cobalt-based materials and effectively improving the water-splitting performance issues of CuCo_2_S_4_.^28,29^ Subsequent fluorination treatments are considered feasible methods for regulating electronic structures and optimizing the absorption of active components. Therefore, introducing V, Co, F can effectively cause structural changes and synergistic effects, enhancing the activity of the catalyst and accelerating its kinetics.^30,31^ For instance, Tao *et al.*^32^ synthesized cobalt–vanadium-based nanocomposites *via* a one-step hydrothermal method, achieving a current density of 10 mA cm^−2^ at overpotentials of 210 mV for the oxygen evolution reaction and 130 mV for hydrogen generation. Kadam *et al.*,^33^ through hydrothermal synthesis of bimetallic cobalt–vanadium oxide Co_3_V_2_O_8_ in 1 M KOH electrolyte, achieved a hydrogen evolution reaction of 226 mV and a Tafel slope of 178 mV dec^−1^.

Inspired by the above, we adopted a multi-step hydrothermal synthesis and (under argon protection) fluorination treatment to prepare CuCo_2_S_4_@Co–V–O–F catalysts with nanowire arrays. Through the synergistic effect of Cu and Co, high-valence V providing additional redox centers, and surface modification by fluorination, its surface chemistry was altered to promote the adsorption and dissociation of water molecules, thereby improving the performance and stability issues of the CuCo_2_S_4_ matrix. The prepared CuCo_2_S_4_@Co–V–O–F catalyst exhibits excellent performance in hydrogen evolution reaction. In 1 M alkaline KOH solution, it shows a low overpotential of 87.8 mV to achieve a current density of −10 mA cm^−2^ and a Tafel slope of 176.7 mV dec^−1^. Meanwhile, in 1 M alkaline seawater KOH solution, the HER performance only shows an overpotential of 95.5 mV, which is merely 7.7 mV higher than that in KOH solution, with a Tafel slope of 149.46 mV dec^−1^. After 24 hours of cycling, the hydrogen evolution overpotential surprisingly decreases from the original 225.7 mV (−50 mA cm^−2^) to 213.6 mV. In terms of OER performance, it also decreases from 227.3 mV in KOH solution to 212.3 mV to achieve a current density of 10 mA cm^−2^ and has a low cell voltage of 1.543 V when electrolyzing seawater at 50 mA cm^−2^. Notably, regarding the double-layer capacitance (*C*_dl_) corresponding to active sites for hydrogen evolution reactions, it performs excellently, reaching 168.56 mF cm^−2^ and 208.95 mF cm^−2^ respectively. This study provides a beneficial guiding direction for exploring bifunctional catalysts with higher activity and greater stability.

## 2. Experimental section

### 2.1. Materials

Cupric nitrate trihydrate [Cu(NO_3_)_2_·3H_2_O, 99%], cobalt(ii) nitrate hexahydrate [Co(NO_3_)_2_·6H_2_O, 99%], ammonium metavanadate [NH_4_VO_3_], ammonium fluoride (NH_4_F, 96%), urea (H_2_NCONH_2_, 99.5%), sodium sulfide nonahydrate (Na_2_S·9H_2_O, 98%), potassium hydroxide (KOH), anhydrous ethanol (C_2_H_6_O, 99.5%), hydrochloric acid (HCl), and nickel foam (Ni foam) were all analytical grade chemicals purchased from Sigma-Aldrich Chemical Company without the need for further purification. All catalysts' hydrothermal synthesis was carried out in a stainless steel autoclave with a polytetrafluoroethylene liner.

### 2.2. Preparation of CuCo_2_S_4_ nanosheets

First, the nickel foam was pre-treated by immersing it in 0.1 M hydrochloric acid for 1 hour. After removal, it was cleaned in an ultrasonic cleaner, rinsed three times with deionized water, and then dried in an oven at 60 °C for 12 hours.

**Scheme 1:**
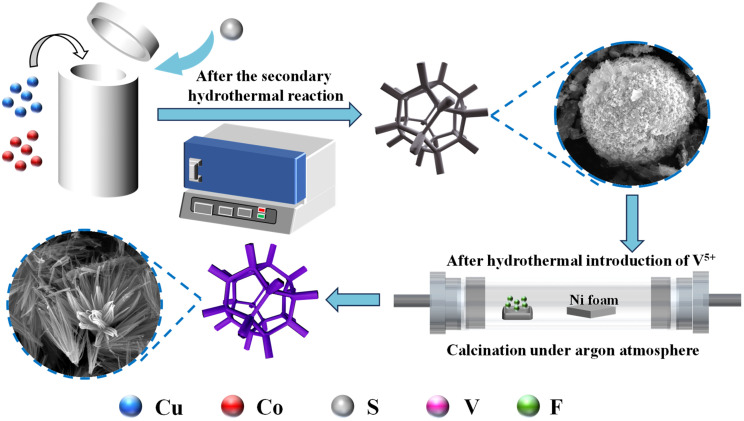
Schematic illustration of synthesizing of CuCo_2_S_4_@Co–V–O–F.1 foam nickel.

As shown in the formation steps in Scheme 1, a two-step hydrothermal method was used to prepare the CuCo_2_S_4_ matrix. A solution was prepared by dissolving 4 mM Cu(NO_3_)_2_·3H_2_O, 4 mM Co(NO_3_)_2_·6H_2_O, 4 mM NH_4_F, and 3 mM urea in 70 mL of deionized water under stirring for 45 minutes. The pre-treated nickel foam (4 × 4 cm) and the above solution were then placed into a 100 mL autoclave and maintained at 150 °C for 7 hours. After naturally cooling to room temperature, the prepared precursor was washed with anhydrous ethanol and deionized water, and dried at 60 °C for more than 12 hours. Subsequently, a sulfidation treatment was carried out: 0.35 g of Na_2_S·9H_2_O was dissolved in 60 mL of deionized water, mixed with the previously prepared nickel foam, and placed in the autoclave at 120 °C for 4 hours. After natural cooling to room temperature, the same washing and drying procedures were followed. The resulting matrix was named CuCo_2_S_4_ and served as the precursor for subsequent treatments.

A one-step hydrothermal synthesis was performed on the precursor as follows: 0.045 g Co(NO_3_)_2_·6H_2_O; 0.009 g NH_4_VO_3_; 0.045 g Co(NO_3_)_2_·6H_2_O; 0.018 g NH_4_VO_3_; 0.045 g Co(NO_3_)_2_·6H_2_O, along with 4 mM NH_4_F and 3 mM urea, were added to the autoclave and maintained at 120 °C for 4 hours. After natural cooling to room temperature, the product was washed and dried. The obtained product was transferred to ammonium fluoride (15 mg) and heated to 450 °C at a rate of 2.5 °C min^−1^ under an argon atmosphere for 1 hour. After furnace cooling to room temperature, the samples were collected and named CuCo_2_S_4_@CoF_2_, CuCo_2_S_4_@Co–V–O–F.1, and CuCo_2_S_4_@Co–V–O–F.2, respectively.

### 2.3. Material characterization and electrocatalytic performance characterization

The crystal structure of the prepared samples was characterized by X-ray diffraction (XRD, Shimadzu-7000, Cu Kα) in the range of 10–90° at a scanning speed of 8° min^−1^. The surface chemical states, elemental valence states, and qualitative and quantitative information of the elements were determined by X-ray photoelectron spectroscopy (XPS, ESCALAB 250 with an Al Kα source). The microscopic morphological changes of the prepared samples were characterized by scanning electron microscopy (SEM, Gemini 300-71-31).

All electrochemical tests were performed on a CHI760E electrochemical workstation (Chenhua Instruments). Tests were conducted in 1 M KOH (pH = 13.7) and 1 M KOH (pH = 13.51) alkaline seawater solutions as electrolytes. In a conventional three-electrode system, Hg/HgO was used as the reference electrode, graphite rod was used as the counter electrode for HER performance evaluation, while a platinum (Pt) electrode served as the counter electrode during OER testing, and the prepared samples (0.5 cm × 0.5 cm) as the working electrodes for electrochemical testing. Linear sweep voltammetry (LSV) curves were corrected with 90% IR compensation and converted to the reversible hydrogen electrode (RHE) scale using the Nernst equation: *E*_RHE_ = *E*_Hg/HgO_ + 0.059 × pH + 0.098. The OER overpotential was calculated using *η* = *E*_RHE_ − 1.23 V.

## 3. Results and discussion

**Fig. 1:**
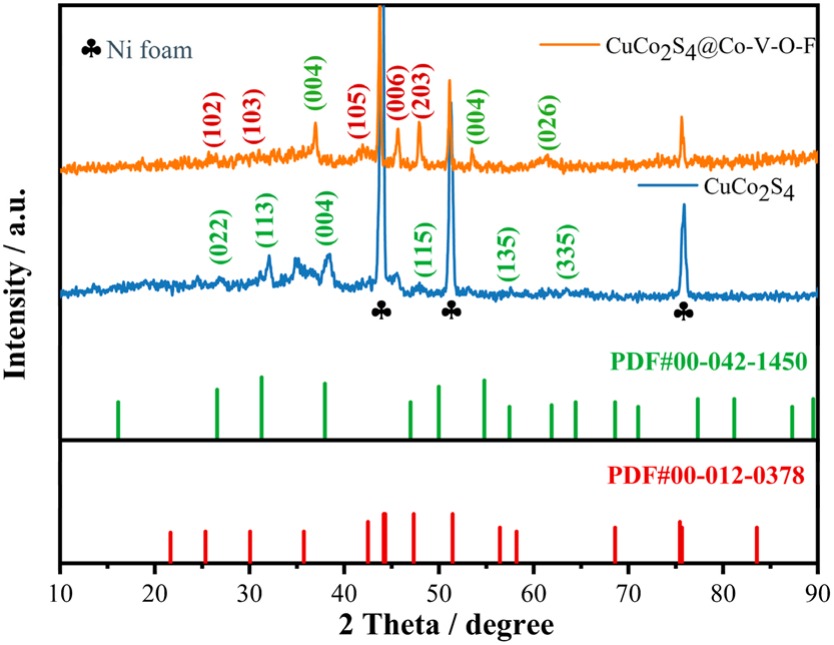
XRD pattern.

First, the phase composition of the prepared samples was characterized by X-ray diffraction (XRD). As shown in Fig. 1, the strong diffraction peaks observed at 2*θ* values of 44.4°, 51.6°, and 76.1° correspond to the reflections of the nickel foam substrate (JCPDS No. 04-0850). The CuCo_2_S_4_ matrix exhibits diffraction patterns that are highly consistent with the standard cubic crystal structure of CuCo_2_S_4_ (PDF# 00-042-1450), indicating that the sulfidation treatment successfully yielded the desired crystalline phase. The characteristic peaks at approximately 26.5°, 31.2°, 37.9°, and 63.7° can be indexed to the (022), (113), (004), and (335) crystal planes, respectively. For the composite sample CuCo_2_S_4_@Co–V–O–F, strong diffraction peaks corresponding to Co and V are observed at 25.9°, 30.3°, 45.4°, and 47.7°, which match well with the (102), (103), (006), and (203) planes of Co_3_V (PDF# 00-012-0378). Notably, the lattice parameters undergo changes due to additional strain introduced after compositing, resulting in lattice distortion. This is manifested as a slight shift of the (004) plane to the left by 0.8°. Furthermore, no distinct characteristic peaks attributable to fluorine (F) or fluoride compounds are observed in the XRD pattern, suggesting that F may exist on the catalyst surface in the form of highly dispersed atoms or small clusters, with either size or concentration below the detection limit of XRD. The absence of detectable F-related peaks also indicates effective dispersion and incorporation of fluorine into the catalyst, which may enhance its stability and activity without altering the overall crystal structure.

**Fig. 2:**
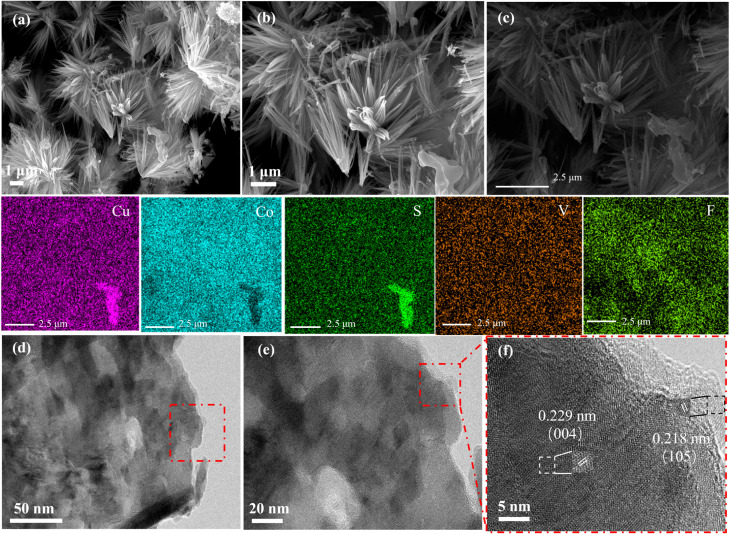
Morphology and structure characterization of the as-prepared products. (a and b) SEM images of CuCo_2_S_4_@Co–V–O–F.1 (c) EDS elemental mapping of Cu, Co, S, V and F for CuCo_2_S_4_@Co–V–O–F.1. (d and e) TEM images of CuCo_2_S_4_@Co–V–O–F.1 samples. (f) HRTEM image of the CuCo_2_S_4_@Co–V–O–F.1 composite.

SEM was used to characterize the microstructural changes of the CuCo_2_S_4_ matrix and its composite materials. The SEM images in Fig. S1(a and b)† show that the CuCo_2_S_4_ matrix consists of coral-like nanoblocks forming nanospheres. After compositing with CoF_2_ material, the morphology transforms into a structure with more nanowires (Fig. S1(c and d)†). The formation of these nanowires increases the contact area between the electrolyte and the catalyst, which positively impacts the kinetics of the electrocatalytic reaction. This is one of the main reasons for the improved electrocatalytic performance of the CuCo_2_S_4_@CoF_2_ sample. However, under high magnification, some incompletely transformed nanodendrites are still observed, appearing thin, poorly defined, and uneven. In the high- and low-magnification images of Fig. 2a and b, the nanowire arrays of the CuCo_2_S_4_@Co–V–O–F.1 sample show significantly more uniform and complete transformation, with the nanowires extending outward from their centers. These peripheral fine nanowire structures contribute positively to charge transfer. At the same time, as a substrate, nickel foam provides a significant advantage for the uniform loading of CuCo_2_S_4_@Co–V–O–F active components, largely due to its three-dimensional porous structure and stable 3D skeletal framework. This unique structure not only ensures excellent structural stability but also enhances the adhesion between the catalyst and the substrate. The large specific surface area and good conductivity of nickel foam contribute to improving the dispersion of CuCo_2_S_4_@Co–V–O–F, further enhancing electron transfer performance and increasing the density of exposed catalytic active sites. Moreover, the strong interaction at heterojunction interfaces effectively boosts the adhesion between the catalyst and the substrate, preventing detachment or deactivation during long-term reactions. This combination of properties makes nickel foam an ideal choice as a substrate material, significantly contributing to the overall efficiency and durability of the catalytic system. Fig. 2c shows the elemental mapping of CuCo_2_S_4_ @Co–V–O–F.1, where the presence of Cu, Co, S, V, and F elements is identified, demonstrating their uniform distribution. Fig. 2d and e present the TEM images of the CuCo_2_S_4_@Co–V–O–F.1 composite material, revealing its overall structure. The surface morphology shows no significant aggregation, nanoscale pores, or voids, indicating that the fluorine (F) surface treatment effectively enhances the dispersion of the material. Fig. 2f provides a high-resolution TEM (HRTEM) image that clearly demonstrates the core–shell structure of the CuCo_2_S_4_@Co–V–O–F.1 composite. The Co–V–O–F layer uniformly coats the CuCo_2_S_4_ substrate at the nanoscale, forming a well-defined core–shell architecture. The lattice fringes are clearly visible in the HRTEM image, with interplanar distances corresponding to the (004) plane of CuCo_2_S_4_ and the (105) plane of Co–V–O–F measured as 0.229 nm and 0.218 nm, respectively. These values are consistent with the XRD analysis results shown in Fig. 1, further confirming the structural characteristics of the composite material. This clear and ordered crystalline arrangement supports the successful formation of the core–shell structure and highlights the precise control over the material's nanoscale design.

**Fig. 3:**
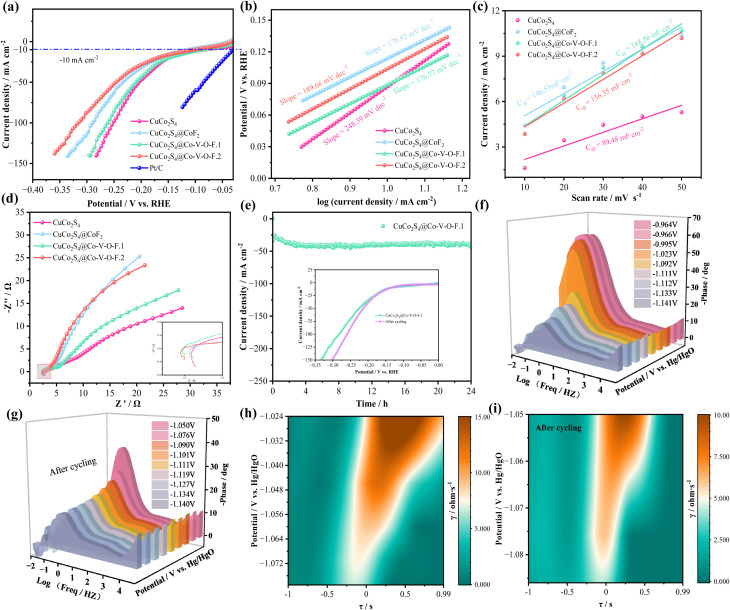
HER performances in 1.0 M KOH solutions. (a) Polarization curves at scan rate of 5 mV s^−1^. (b) Tafel plots. (c) CV curves of double-layer capacitance (*C*_dl_). (d) Nyquist plots. (e) Chronoamperometric stability tests and the insets are LSV curves. (f and g) Nyquist plots at multiple voltages. (h and i) DRT spectrum of CuCo_2_S_4_@Co–V–O–F.1 sample before and after cycling.

In a three-electrode system, Fig. 3 shows the HER performance of various samples in 1.0 M KOH alkaline electrolyte. As shown in Fig. 3a, the sample CuCo_2_S_4_@Co–V–O–F.1 exhibits a lower overpotential (87.8 mV at −10 mA cm^−2^) compared to CuCo_2_S_4_, CuCo_2_S_4_@CoF_2_, and CuCo_2_S_4_@Co–V–O–F.2, whereas the overpotential for the precious metal Pt/C catalyst is 44.2 mV at −10 mA cm^−2^. Kinetic factors cannot be ignored; the Tafel slope value reflects the kinetics of water splitting. In Fig. 3b, the Tafel slope for CuCo_2_S_4_@Co–V–O–F.1 is 176.77 mV dec^−1^, significantly lower than that of CuCo_2_S_4_ (248.39 mV dec^−1^), CuCo_2_S_4_@CoF_2_ (176.92 mV dec^−1^), and CuCo_2_S_4_@Co–V–O–F.2 (189.66 mV dec^−1^). A lower Tafel slope has a positive effect on the kinetic rate control. To discuss the issue of catalyst activity, double-layer capacitance (*C*_dl_) was used to elucidate the electrochemical active surface area (ECSA). As shown in Fig. 3c, CuCo_2_S_4_@Co–V–O–F.1 exhibits a relatively high ECSA of 168.56 mF cm^−2^ compared to other catalysts. Fig. 3d shows that the sample CuCo_2_S_4_@Co–V–O–F.1 has a smaller semicircle diameter in the high-frequency region of the curve, which is closely related to the charge transfer rate at the electrode surface. The smaller the diameter, the lower the charge transfer impedance, and the faster the kinetics of the electrochemical reaction. In the low-frequency region, reference can be made to the following formula:^34^

1 *Z* = *R*_s_ + *R*_ct_ + *σ*_w_*ω*^−1/2^

Among which, *σ*_w_ represents the Warburg factor, *ω* represents the angular frequency. *Z* is attributed to the diffusion resistance of OH^−^. Subsequently, the cyclic stability of the CuCo_2_S_4_@Co–V–O–F.1 catalyst in HER performance was tested (Fig. 3e), finding that after 24 hours, the current decay rate was slow. The inset shows a comparison of LSV curves before and after cycling, indicating that after cycling, there is a trend of decreased overpotential, highlighting the good stability of the catalyst. These excellent HER performances are all benefited from the change in micro-morphology with the addition of nanowires. Fig. 3f shows the three-dimensional impedance plot (Bode plot) of CuCo_2_S_4_@Co–V–O–F.1 at various voltages corresponding to −10 mA cm^−2^, −20 mA cm^−2^ up to −100 mA cm^−2^. The phase angle *versus* frequency curve reflects the dynamic response of the catalyst sample.^35^ The figure indicates that as the applied potential increases, the phase angle in the low-frequency region decreases orderly, showing a continuous decrease in charge transfer resistance (*R*_ct_), which results in an increasing charge transfer rate. The Bode plot after cycling (Fig. 3g) shows that the phase angles after cycling are more regularly arranged, further highlighting the excellent charge transfer rate of CuCo_2_S_4_@Co–V–O–F.1. Fig. 3h presents the Distribution of Relaxation Times (DRT) map for CuCo_2_S_4_@Co–V–O–F.1, providing further explanation of polarization impedance. The horizontal axis represents relaxation time (*τ*), representing the frequency range; the left vertical axis shows the voltage applied corresponding to gradually increasing current density; the color gradient on the right vertical axis *γ*/ohm·s^−1^ represents the distribution function of relaxation times (*i.e.*, the contribution of the polarization process to total impedance).^36,37^ As shown, with the continuous increase in applied voltage, the color of characteristic peaks gradually fades, indicating a decreasing contribution of polarization impedance, thereby accelerating charge transport. The kinetics of the diffusion process is negatively correlated with the polarization impedance value. At the same time, in the high-frequency region, the relaxation time *τ* corresponds to rapid electrochemical processes (such as charge transfer, interfacial polarization), typically *τ* < 10^−1^ seconds. In this region, the kinetics of the diffusion process are less likely to be impeded by impedance.^38,39^ Therefore, the DRT plot after cycling (Fig. 3i) not only shows a reduction in the maximum impedance contribution but also an overall shift towards the high-frequency region (to the left), with characteristic peaks moving towards higher frequencies, and the interval corresponding to *τ* has been reduced, indicating a decrease in peak area. All these indicate a reduction in electrode interface charge transfer resistance and polarization impedance, thus accelerating the charge transfer process.

**Fig. 4:**
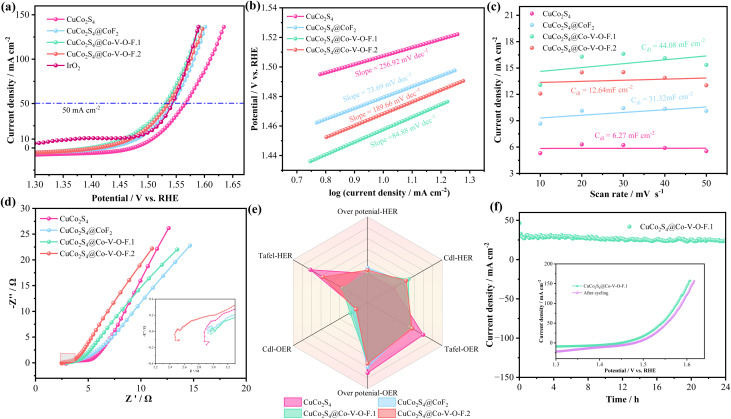
OER performances in 1.0 M KOH solutions. (a) Polarization curves at scan rate of 2 mV s^−1^. (b) Tafel plots. (c) Double-layer capacitance (*C*_dl_). (d) Nyquist plots. (e) Radar chart. (f) Chronoamperometric stability tests the insets is LSV curves before and after cycling.

The LSV curves at a scan rate of 2 mV s^−1^ for the prepared samples indicate that, at a current density of 50 mA cm^−2^, the overpotential for the CuCo_2_S_4_@Co–V–O–F.1 sample is 299.3 mV, which is higher than that of the precious metal IrO_2_ (289 mV) but still advantageous compared to CuCo_2_S_4_ (335.3 mV), CuCo_2_S_4_@CoF_2_ (313.3 mV), and CuCo_2_S_4_@Co–V–O–F.2 (306.7 mV). Moreover, the overpotential for a Ni substrate at the same current density is 450 mV, indicating its negligible contribution to electrocatalytic performance. The Tafel plot in Fig. 4b explores the reaction kinetics of the samples, showing that CuCo_2_S_4_@Co–V–O–F.1 has a low Tafel slope of 84.88 mV dec^−1^, suggesting efficient reaction kinetics. Fig. 4c compares the *C*_dl_ (double-layer capacitance) values of the electrocatalysts, from which the activity order can be deduced as CuCo_2_S_4_@Co–V–O–F.1 > CuCo_2_S_4_@CoF_2_ > CuCo_2_S_4_@Co–V–O–F.2 > CuCo_2_S_4_. Higher *C*_dl_ values indicate larger active surface areas of the electrocatalyst, providing more active sites for the electrocatalytic reaction. Fig. 4d shows the EIS (Electrochemical Impedance Spectroscopy) spectra, where the CuCo_2_S_4_@Co–V–O–F.1 sample exhibits a smaller semicircle diameter in the high-frequency region, indicating lower impedance during charge transfer. The radar chart in Fig. 4e showcases important parameters related to HER and OER performance for the four mentioned products, highlighting that CuCo_2_S_4_@Co–V–O–F.1 performs better overall. The prominence in HER-*C*_dl_ and OER-*C*_dl_ parts, along with other parameter sections, underscores its comprehensive superiority. Introducing high-valence V into the matrix enhances the OER performance due to its high oxidation state,^33^ enabling participation in more redox reactions and improving electron transfer efficiency between the catalyst and oxygen intermediates, thus lowering the energy barrier in the OER process and boosting catalytic activity. A 24-hour long cycling test (Fig. 4f) and the comparison of LSV curves before and after cycling demonstrate good cyclic stability. Table 1 compares the electrocatalytic performance of CuCo_2_S_4_@Co–V–O–F.1 with previously reported results, emphasizing the superior electrocatalytic performance of this sample.

**Table 1:** Comparison of electrocatalytic performance of CuCo_2_S_4_@Co–V–O–F.1 with previous literature reports

Materials	Performances	Electrolyte	*η* (mV)	Ref.
CuCo_2_S_4_@Co–V–O–F.1	HER	1 M KOH	87.8 mV (10 mA cm^−2^)	This work
	OER		227.3 mV (10 mA cm^−2^)	
CuCo_2_S_4_@Ni(OH)_2_	HER	1 M KOH	117 mV (10 mA cm^−2^)	47
(Co,V)-FeOOH	OER	1 M KOH	227.5 mV (10 mA cm^−2^)	48
CuCo_2_S_4_@CoS_2_	HER	1 M KOH	153 mV (10 mA cm^−2^)	49
	OER		261 mV (10 mA cm^−2^)	
Co/VN	HER	1 M KOH	92 mV (10 mA cm^−2^)	50
Co_2_VO_4_	OER		300 mV (10 mA cm^−2^)	
CVO	HER	1 M KOH	91.4 mV (10 mA cm^−2^)	51
CoVFeN	OER	1 M KOH	264 mV (100 mA cm^−2^)	52

**Fig. 5:**
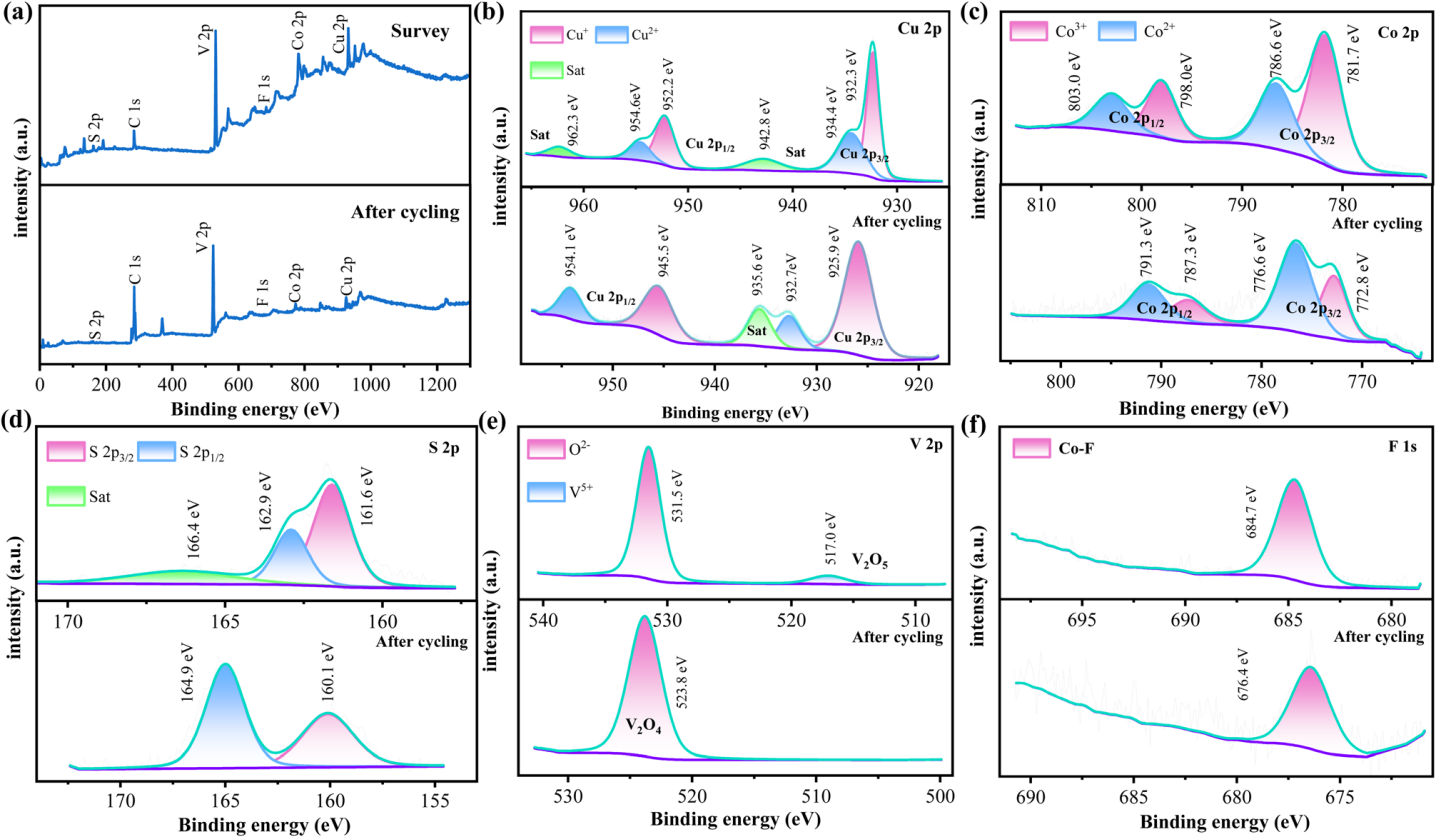
(a) The high resolution XPS Survey spectra before and after cycling. (b) XPS of Cu 2p (c) Co 2p (d) S 2p (e) V 2p (f) F 1s.

Based on previous work, XPS was used to analyze the surface chemical states and electronic structures of the CuCo_2_S_4_@Co–V–O–F.1 sample before and after OER cycles. Elemental composition CuCo_2_S_4_@Co–V–O–F.1 As shown in the percentage content Graph S2,† the sample indicates the presence of carbon C, V, Cu, Co, S, and F, with vanadium accounting for 27.9% of the composition, indicating its supporting role in the material. Fig. 5a compares the full spectra before and after cycling, revealing a significant increase in the C 1s peak, while other elements showed a slight decrease, mainly due to the minor precipitation of reactants during testing, leading to a reduction in their concentration. Fig. 5b shows the Cu 2p spectrum of the prepared sample, where two peaks at 932.3 eV and 952.2 eV correspond well with the spin–orbit doublets of Cu 2p_3/2_ and Cu 2p_1/2_.^40^ In the Cu 2p spectrum, the characteristic peaks fitted at 932.3 eV and 952.2 eV can be assigned to Cu^2+^, while two peaks near 934.4 eV and 954.6 eV are attributed to the presence of Cu^+^.^41^ Additionally, two peaks around 942.8 eV and 962.3 eV can be attributed to shake-up satellites (denoted as Sat.). The post-cycling Cu 2p spectrum shows an increased FWHM for Cu^+^ from 1.42 eV to 3.27 eV, indicating hydroxylation and changes in elemental content, suggesting exposure of more coordination sites. Meanwhile, there is a noticeable decrease in the satellite peak of Cu^2+^ at Cu 2p_3/2_, likely related to changes in oxidation state and chemical environment, which decreases with consumption during testing, but the energy difference between the main peaks remains approximately 20 eV without significant change. Fig. 5c depicts the pre- and post-cycling spin–orbit spectra of Co 2p. In the upper figure, two peaks at around 798 eV and 781.7 eV correspond to Co 2p_1/2_ and Co 2p_3/2_ states, respectively. Moreover, peaks observed at 803 eV and 786.6 eV confirm the presence of divalent Co, while peaks at 798 eV and 781.7 eV indicate the presence of Co^3+^. The ratio of Co^3+^ (45.69%)/Co^2+^ (23.95%) for Co 2p_3/2_ exceeds 1.9, and the Co^3+^ (17.36%)/Co^2+^ (13.00%) ratio for Co 2p_1/2_ exceeds 1.3, indicating the presence of oxygenated surfaces and Co^2+^ oxidation states.^42,43^ During CO oxidation reactions, the surface typically undergoes dynamic reduction (CO(iii) → CO(ii)) and reoxidation (CO(ii) → CO(iii)) cycles. The reduction in Co^3+^ content in the post-cycling Co 2p spectrum, converting to more divalent Co, confirms this. In the S 2p spectrum (Fig. 5d), peaks at 161.6 eV and 162.9 eV can be attributed to S 2p_3/2_ and S 2p_1/2_, respectively, while a broad peak at 166.4 eV is a satellite peak.^44^ An increase and certain shift in S 2p_1/2_ after cycling are explained by strong interactions involving S in adsorption. Fig. 5e analyzes the V 2p fitting curve spectrum, where a peak at 517.00 eV corresponds to the characteristic peak of V^5+^ in the V 2p orbital, consistent with the oxidation state of vanadium in vanadium pentoxide (V_2_O_5_). A peak near 531.50 eV falls within the typical binding energy range for O 1s orbitals (530–535 eV), possibly corresponding to lattice oxygen O^2−^ in V_2_O_5_, whereas a peak appearing at 523.8 eV after cycling can be attributed to V_2_O_4_.^45^ This indicates the presence of different valence states of V in the CuCo_2_S_4_@Co–V–O–F.1 nanowire array, and redox reactions of Co and V in different valence states can accelerate electron transfer, which is one of the main reasons for improved OER performance. The F 1s spectrum in Fig. 5f reveals that the Co–F bond dominates at 684.7 eV,^46^ while the post-cycling binding energy is 676.6 eV, indicating a significant shift in binding energy due to interactions between fluorine and other elements, proving effective doping of the F element and its contribution to the sample's reaction.

In summary, we have successfully prepared the CuCo_2_S_4_@Co–V–O–F.1 catalyst using a multi-step hydrothermal process followed by calcination under argon protection. This catalyst exhibits excellent performance in both hydrogen evolution reaction and oxygen evolution reaction in alkaline electrolytes, showing low overpotentials of 87.8 mV for HER and 227.3 mV for OER at −10 mA cm^−2^. Currently, most research on hydrogen production *via* water electrolysis focuses on the use of high-purity freshwater. However, freshwater resources account for only 3.5% of the world's total water resources, and as these resources become increasingly scarce, exploring alternative water sources has become particularly important. The ocean is almost an inexhaustible resource, with its contained hydrogen energy potentially capable of meeting future human energy needs.^53^ Therefore, hydrogen production through seawater electrolysis represents a highly promising approach. We evaluated the electrocatalytic activity and stability of the sample in a simulated seawater environment, specifically in 1 M seawater alkaline conditions. This work not only demonstrates the potential of utilizing seawater for hydrogen production but also highlights the adaptability and stability of the CuCo_2_S_4_@Co–V–O–F.1 catalyst under different environmental conditions.

**Fig. 6:**
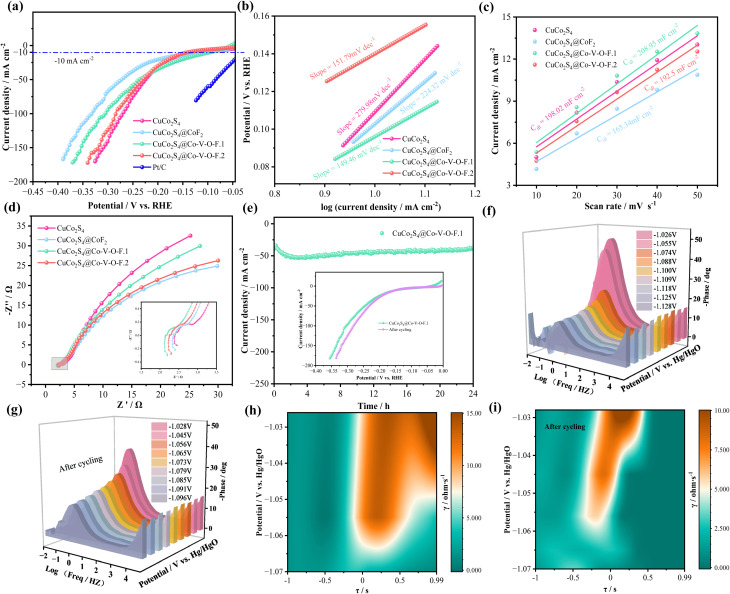
Electrocatalytic performances of the electrocatalysts in alkaline seawater. (a) LSV curves for HER. (b) Tafel plots. (c) Double-layer capacitance (*C*_dl_). (d) Nyquist plots. (e) Chronoamperometric stability tests and the insets are LSV curves. (f and g) Nyquist plots at multiple voltages. (h and i) DRT spectrum of CuCo_2_S_4_@Co–V–O–F.1 sample before and after cycling.

For the evaluation of the electrocatalytic performance of the samples in seawater, the HER performance of the electrocatalyst was first tested in a 1.0 M KOH seawater alkaline electrolyte. As shown in Fig. 6a, CuCo_2_S_4_@Co–V–O–F.1 exhibited an overpotential of 95.5 mV at a current density of −10 mA cm^−2^, which is lower than that of CuCo_2_S_4_ (108.5 mV), CuCo_2_S_4_@CoF_2_ (103.5 mV), and CuCo_2_S_4_@Co–V–O–F.2 (139.1 mV). To assess the catalytic reaction kinetics for all catalysts, the corresponding Tafel slopes were calculated from LSV curves. Fig. 6b shows that CuCo_2_S_4_@Co–V–O–F.1 has the lowest Tafel slope of 149.46 mV dec^−1^ compared to other samples, indicating outstanding kinetics and excellent catalytic activity. The electrochemical active surface area (ECSA) was used to study the catalytic activity of the catalysts, and the *C*_dl_ values were plotted for illustration. The slope value for CuCo_2_S_4_@Co–V–O–F.1 was 208.95 mF cm^−2^, higher than those of CuCoS_4_ (198.02 mF cm^−2^), CuCo_2_S_4_@CoF_2_ (165.34 mF cm^−2^), and CuCo_2_S_4_@Co–V–O–F.2 (192.5 mF cm^−2^). According to EIS analysis (Fig. 6d), in the Nyquist plot, the diameter of the semicircle in the high-frequency region generally represents *R*_ct_. It can be seen that the CuCo_2_S_4_@Co–V–O–F.1 electrode material has a slight advantage but no significant difference compared with other samples, which can be further analyzed through subsequent impedance diagrams. The cyclic stability test shown in Fig. 6e found that after 24 hours, the sample still maintained well, which depends on the formation of a surface passivation layer that protects the internal morphology and makes materials less susceptible to corrosion. The comparison of LSV curves in the inset indicates a decrease in overpotential after cycling, demonstrating the excellent stability of the CuCo_2_S_4_@Co–V–O–F.1 catalyst. Subsequently, we described the diffusion behavior and arrangement patterns within the diffusion layer near the electrode surface using Bode plots more intuitively, where increased applied voltage is inversely proportional to charge impedance, indicating reduced ion diffusion resistance of the CuCo_2_S_4_@Co–V–O–F.1 sample. The post-cycling Bode plot (Fig. 6g) showed a better decremental arrangement of phase angles. Combined with Fig. 6h and i showing the DRT spectra of CuCo_2_S_4_@Co–V–O–F.1, it can be seen that after prolonged cycling, there is a shift towards the high-frequency region (to the left), with characteristic peaks also moving, suggesting that after longer stability tests, the CuCo_2_S_4_@Co–V–O–F.1 sample is likely to exhibit lower impedance contributions and charge transfer resistance, which undoubtedly benefits charge transport efficiency. One of the main reasons is that an over-oxidation phenomenon occurred on the material surface during the cycling process. Specifically, the outer layer of the material underwent a certain degree of structural and chemical transformation, forming a passivation interfacial layer composed of CuCo_2_S_4_@Co–V–O–F. This thin and stable surface layer acts as a “protective shell,” effectively preventing further oxidation and corrosion of the inner material. In particular, the Co–V–O–F component is believed to be capable of modulating the electronic structure and providing more active sites, while the CuCo_2_S_4_ core ensures good conductivity and structural support. The synergistic effect between surface oxidation and interface passivation is the key to the material's excellent performance during long-term cycling.^54^

**Fig. 7:**
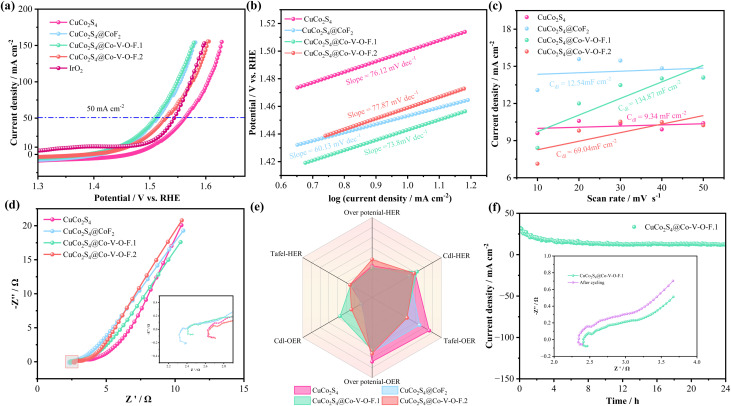
(a) LSV curves for OER. (b) Tafel plots. (c) Double-layer capacitance (*C*_dl_). (d) Nyquist plots. (e) Radar chart. (f) Chronoamperometric stability tests the insets is Nyquist plots before and after cycling.

Regarding the OER-LSV curve in Fig. 7a, it can be observed that at a current density of 50 mA cm^−2^, the overpotential required by the CuCo_2_S_4_@Co–V–O–F.1 sample is 279.1 mV < CuCo_2_S_4_@CoF_2_ (284 mV) < CuCo_2_S_4_@Co–V–O–F.2 (298 mV) < CuCo_2_S_4_ (331 mV). Fig. 7b shows that the Tafel value of CuCo_2_S_4_@Co–V–O–F.1 is 73.8 mV, only slightly higher than that of CuCo_2_S_4_@CoF_2_ (60.13 mV dec^−1^). However, in the *C*_dl_ graph, the value for CuCo_2_S_4_@Co–V–O–F.1 (134.87 mF cm^−2^) is significantly higher than that of CuCo_2_S_4_@CoF_2_ (12.54 mF cm^−2^). Moreover, in the EIS diagram (Fig. 7d), CuCo_2_S_4_@Co–V–O–F.1 exhibits a notably smaller semicircle diameter range in the high-frequency region compared to other samples, indicating good reaction kinetics. Through the above analysis and the more intuitive presentation in the radar chart in Fig. 7e, it can be concluded that the various parameters of the CuCo_2_S_4_@Co–V–O–F.1 sample are clearly superior to those of CuCo_2_S_4_@CoF2, reflecting a faster rate of electrocatalytic reactions overall. The stability test in Fig. 7f demonstrates that the CuCo_2_S_4_@Co–V–O–F.1 sample maintains good stability after 24 hours of long-term cycling, with the charge transfer resistance showing no significant downward trend as indicated in the inset impedance graph.

**Fig. 8:**
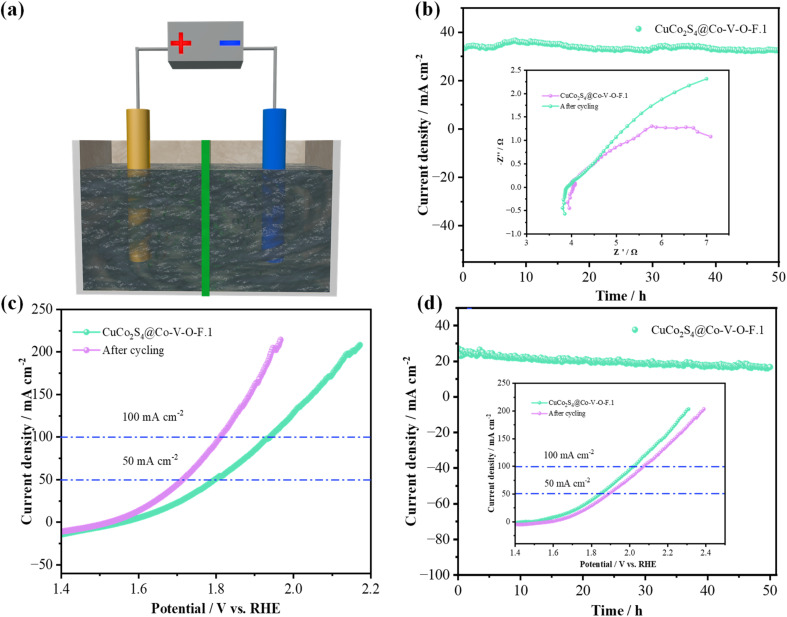
Overall water splitting performance of the electrocatalysts. (a) Device schematic diagram. (b) Overall water splitting stability for CuCo_2_S_4_@Co–V–O–F.1 in 1.0 M KOH. (c) LSV curves. (d) Chronoamperometric stability tests the insets is LSV curve in alkaline seawater.

To further explore the potential application value of the prepared electrocatalysts in overall water splitting, the prepared samples (1.0 cm × 1.0 cm) were assembled at both the anode and cathode of an electrolyzer. Fig. 8b shows that after a 50-hour test in 1.0 M KOH, the corresponding voltage values for the CuCo_2_S_4_@Co–V–O–F.1 sample did not show any significant decrease. Additionally, there was no noticeable difference in impedance in the high-frequency region. Fig. 8c provides a comparison of LSV curves, from which it can be seen that the CuCo_2_S_4_@Co–V–O–F.1 catalyst outputs voltages of 1.796 V and 1.937 V at current densities of 50 mA cm^−2^ and 100 mA cm^−2^, respectively. However, after cycling, the overall overpotential is significantly reduced, indicating that the CuCo_2_S_4_@Co–V–O–F.1 nanowire array exhibits lower cell voltages after prolonged stability tests. The stability test in seawater (Fig. 8d) also demonstrates good cycling life, with the LSV curves after cycling showing that the overpotential does not significantly rise excessively. It confirms that the prepared material possesses excellent structural stability and rapid reaction kinetics during the reaction process. This makes the CuCo_2_S_4_@Co–V–O–F.1 a promising candidate for efficient and stable performance in overall water splitting applications, especially under challenging conditions such as those found in seawater electrolysis.

## 4. Conclusion

In work, we demonstrate the controlled synthesis of CuCo_2_S_4_@Co–V–O–F.1 nanowire array architectures *via* hydrothermal growth and subsequent calcination. The optimized catalyst exhibits superior bifunctional activity across diverse electrolytes, delivering exceptional HER/OER overpotentials of 87.8/227.3 mV at −10 mA cm^−2^ in alkaline freshwater, while maintaining robust performance in simulated seawater (95.5 mV for HER and 213.5 mV for OER at equivalent current density). Morphological optimization coupled with strategic vanadium doping synergistically enhances charge transfer kinetics and stabilizes reactive intermediates during OER processes. A symmetric electrolyzer employing this catalyst achieves an industrially relevant water-splitting voltage of 1.796 V at 50 mA cm^−2^, demonstrating practical viability for large-scale hydrogen generation. This work elucidates structure–activity relationships in transition metal oxy–sulfide hybrids. This indicates significant potential for hydrogen production through water splitting using this material, providing valuable reference and research directions for advancing non-precious metal catalysts.

## Data availability

The data that supports the findings of this study are available from the corresponding authors upon reasonable request.

## Conflicts of interest

The authors declare no conflict of interest.

## Supplementary Material

RA-015-D5RA03052H-s001
