# Supplementary material for: Rapidly reconstructed CuCo2S4@Co–V–O–F nanocatalysts for efficient and stable overall water splitting in alkaline and seawater electrolysis
Source: RSC Adv. 2025 Jun 9;15(24):19443–55. doi: 10.1039/d5ra03052h (PMC12147095; doi:10.1039/d5ra03052h)
Supplement: RA-015-D5RA03052H-s001 [file RA-015-D5RA03052H-s001.pdf]

Supplementary Information (SI) for RSC Advances.
This journal is © The Royal Society of Chemistry 2025

Supporting Information

Rapidly Reconstructed $CuCo_2S_4$@Co-V-O-F Nanocatalysts for Efficient and Stable Overall Water Splitting in Alkaline and Seawater Electrolysis

Boyao Zhang[a], Yinuo Zhao[a], Xin Li[a], Huiya Zhou[a], XinXin Zhao[a], Rongda Zhao[a,*], Fufa Wu[a,*]

[a]School of Materials Science and Engineering, Liaoning University of Technology, Jinzhou, Liaoning 121000, P. R.China

Correspondences addressed: Rongdazhaoln@126.com

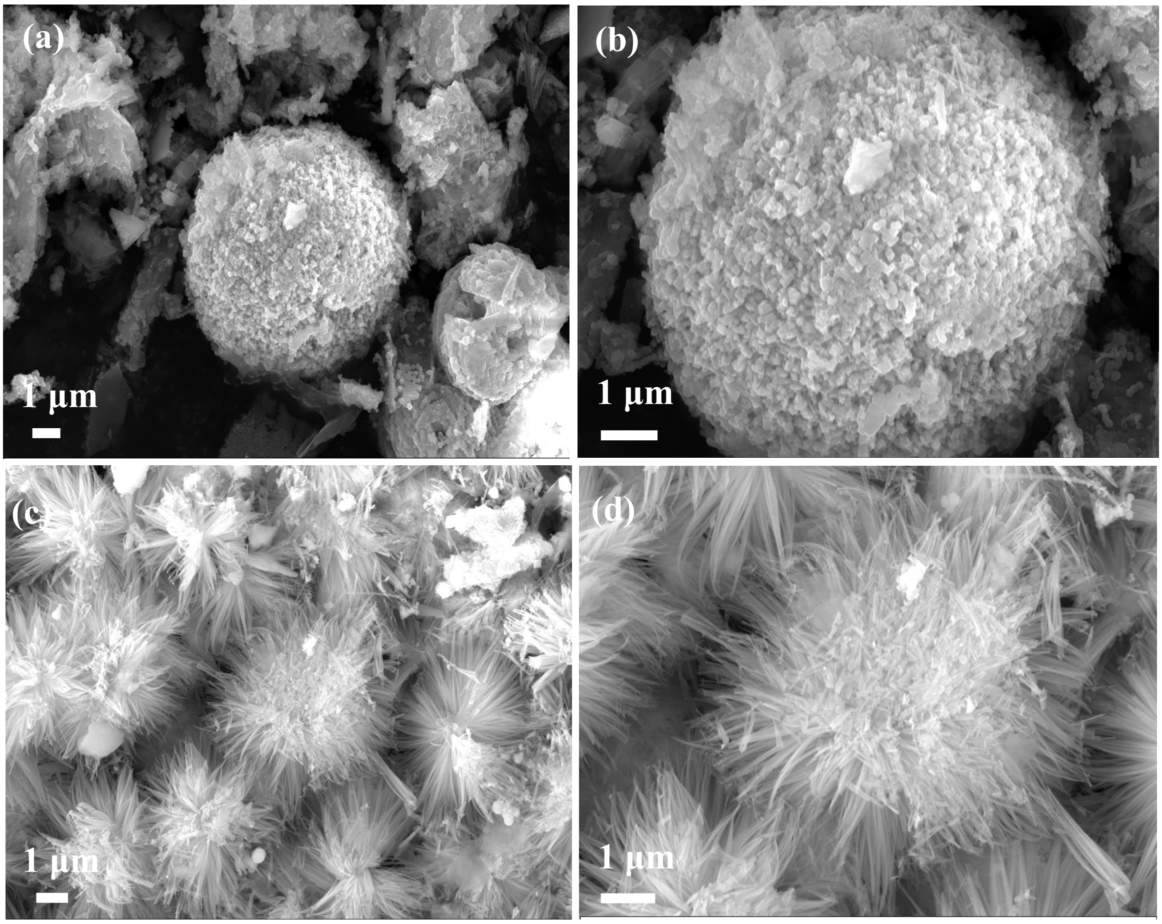

Figure S1 (a,b)SEM images of $CuCo_2S_4$; (c,d) $CuCo_2S_4@CoF_2$.

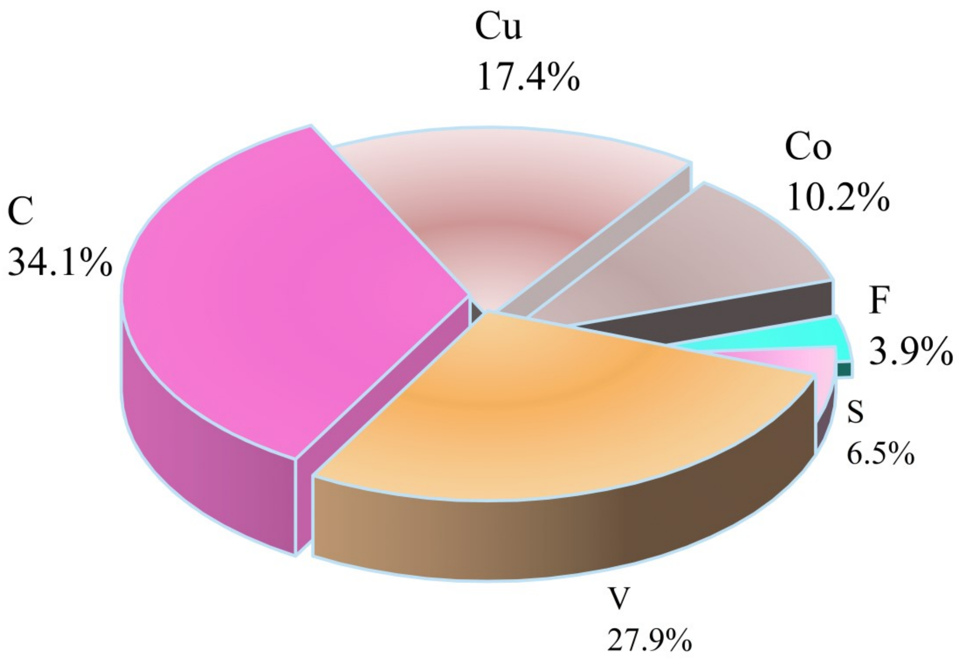

Figure S2 Percentage content chart of $CuCo_2S_4$@ Co-V-O-F.1 sample..

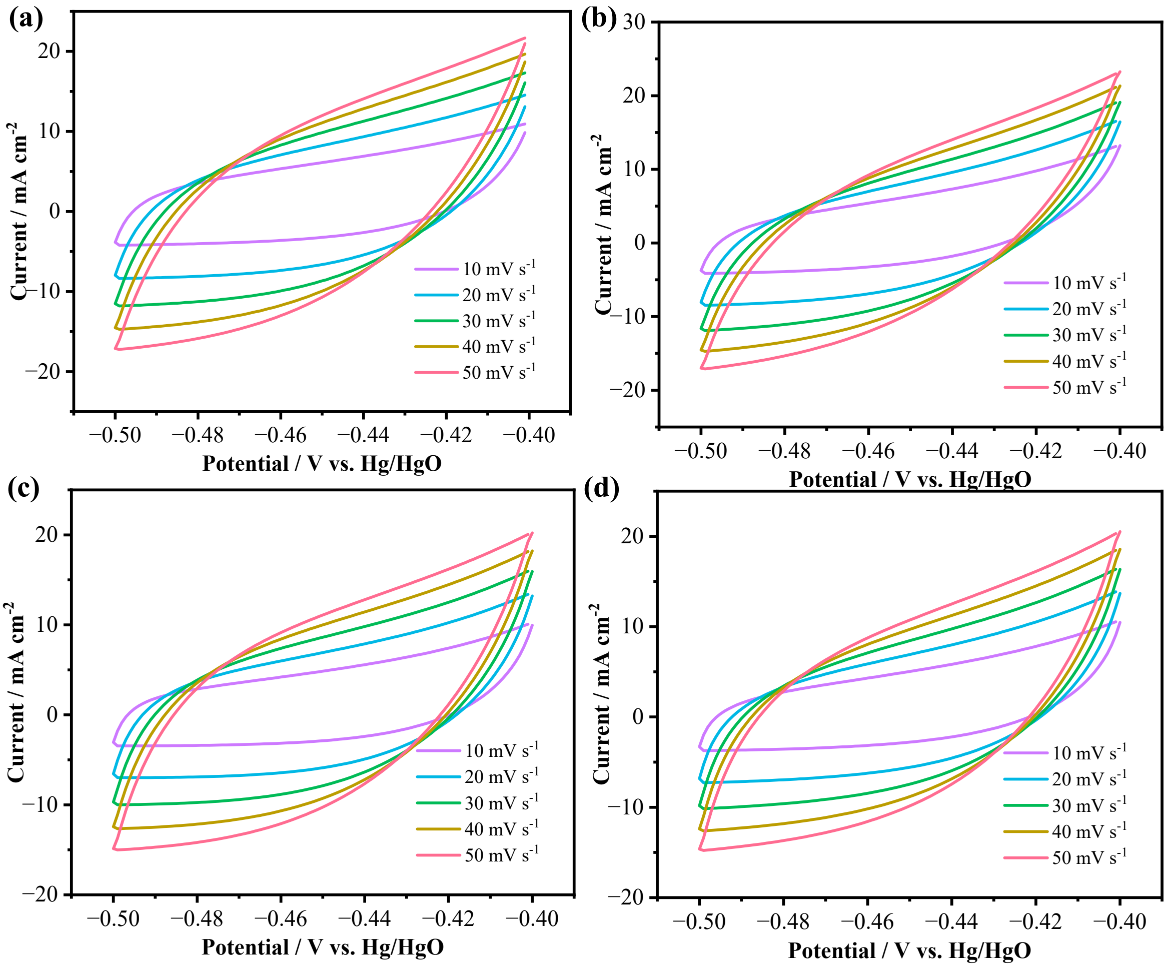

Figure S3 CV curves of HER of the as-obtained samples in KOH solutions; (a) curves of $CuCo_2S_4$; (b) curves of $CuCo_2S_4$@CoF_2; (c) curves of $CuCo_2S_4$@Co-V-O-F-1; (d) curves of $CuCo_2S_4$@Co-V-O-F-2.

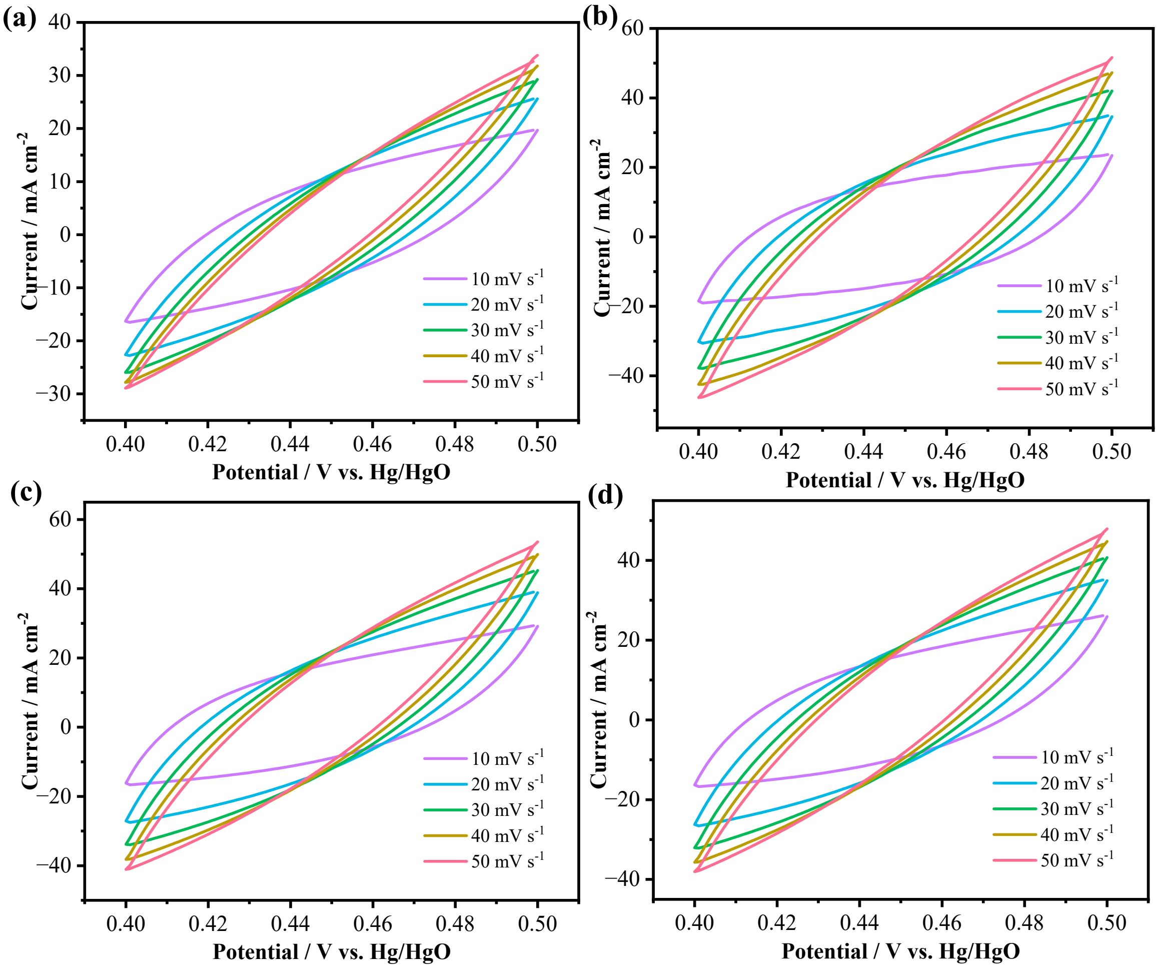

Figure S4 CV curves of OER of the as-obtained samples in KOH solutions; (a) curves of $CuCo_2S_4$; (b) curves of $CuCo_2S_4$@CoF_2; (c) curves of $CuCo_2S_4$@Co-V-O-F_{-1}; (d) curves of $CuCo_2S_4$@Co-V-O-F_{-2}.

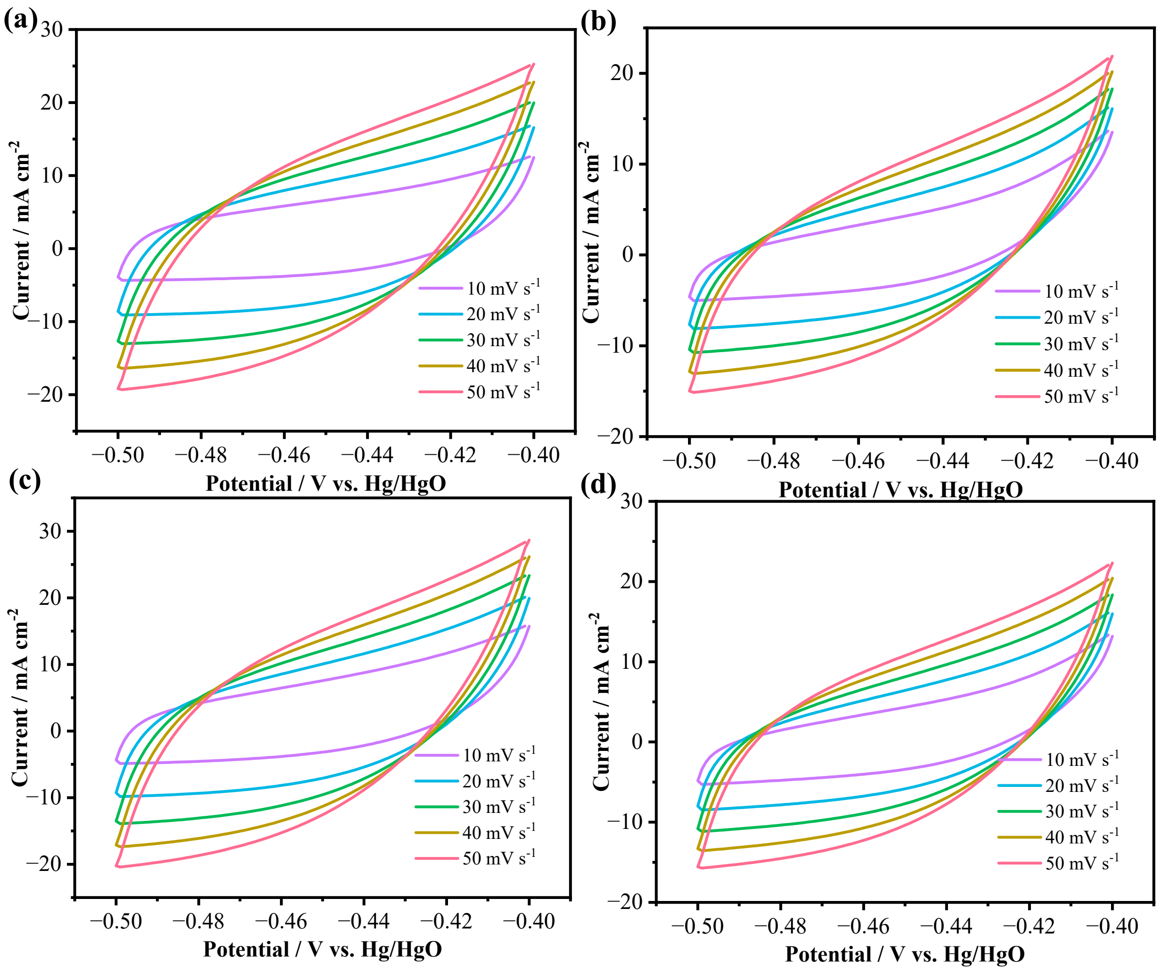

Figure S5 CV curves of HER of the as-obtained samples in seawater; (a) curves of $CuCo_2S_4$; (b) curves of $CuCo_2S_4$@CoF_2; (c) curves of $CuCo_2S_4$@Co-V-O-F$_{-1}$; (d) curves of $CuCo_2S_4$@Co-V-O-F$_{-2}$.

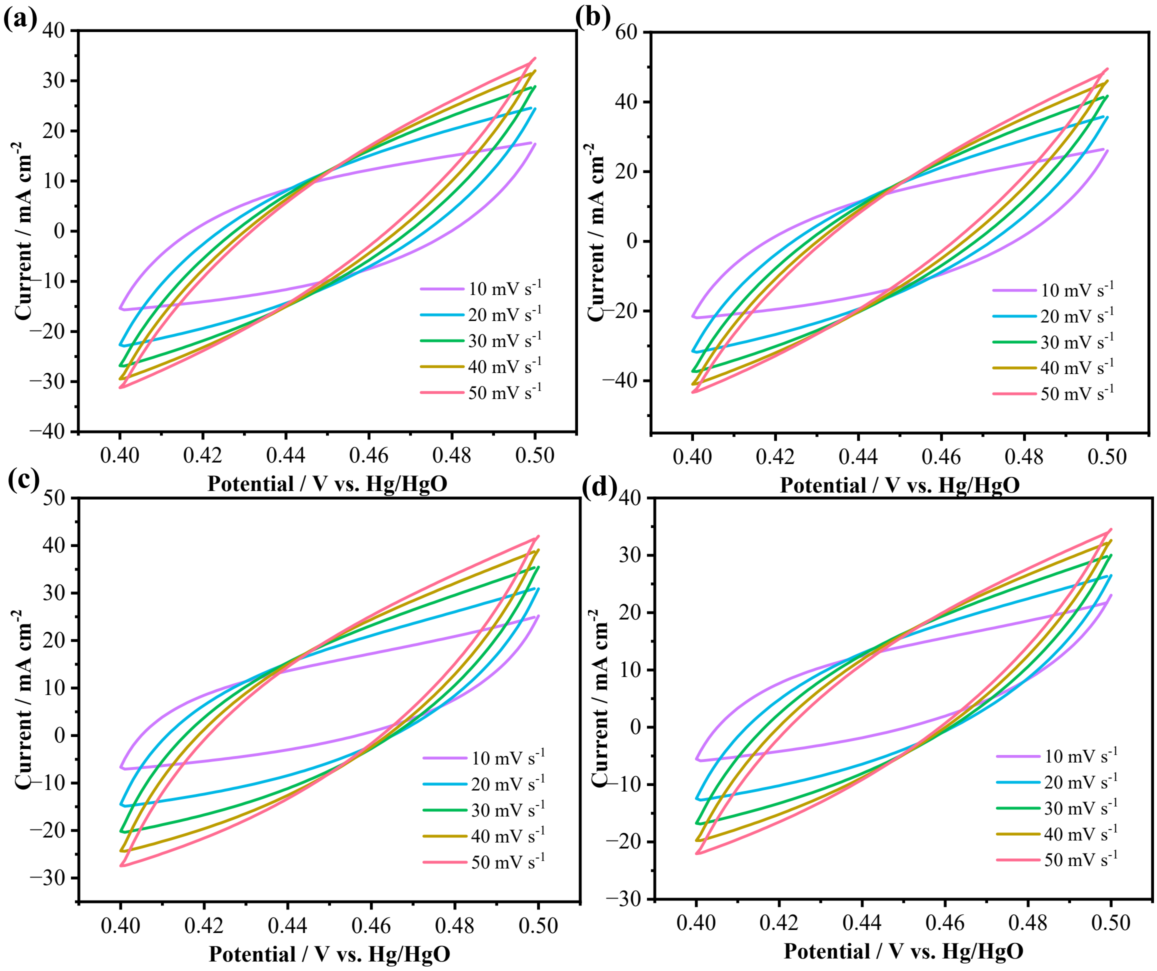

Figure S6 CV curves of OER of the as-obtained samples in seawater; (a) curves of $CuCo_2S_4$; (b) curves of $CuCo_2S_4$@CoF_2; (c) curves of $CuCo_2S_4$@Co-V-O-F$_{-1}$; (d) curves of $CuCo_2S_4$@Co-V-O-F$_{-2}$.
